# Prospects of Future Typhoid and Paratyphoid Vaccines in Endemic Countries

**DOI:** 10.1093/infdis/jiab393

**Published:** 2021-08-10

**Authors:** Mila Shakya, Kathleen M Neuzil, Andrew J Pollard

**Affiliations:** 1 Oxford University Clinical Research Unit—Nepal, Patan Academy of Health Sciences, Lalitpur, Nepal; 2 University of Maryland School of Medicine, Baltimore, Maryland, USA; 3 Oxford Vaccine Group, Department of Paediatrics, University of Oxford, and the National Institute for Health Research Oxford Biomedical Research Centre, Oxford, United Kingdom

**Keywords:** typhoid fever, paratyphoid fever, typhoid conjugate vaccines

## Abstract

Low- and middle-income countries face a high burden of typhoid and paratyphoid fever due to poor water quality and inadequate sanitation. The World Health Organization (WHO) recommends the use of typhoid conjugate vaccines (TCV) in endemic settings and Gavi, the Vaccine Alliance, supports TCV introduction. There are currently 2 WHO-prequalified TCVs with Typbar TCV introduced in Pakistan, Liberia, and Zimbabwe. Countries should assess disease burden and consider introduction of TCV for programmatic use. Several paratyphoid vaccine candidates are in early stages of development. An effective bivalent vaccine would be the most efficient way to control typhoid and paratyphoid fever.

Typhoid and paratyphoid fever, collectively known as enteric fever, are caused by *Salmonella* Typhi (*S*. Typhi) and *Salmonella* Paratyphi (*S*. Paratyphi) A, B, and C. Enteric fever is endemic in low- and middle-income countries that lack sufficient water, sanitation, and hygiene (WASH) infrastructure. While improvements in WASH are the mainstay for control of enteric fever, these long-term investments are often not feasible in many typhoid-endemic countries. Typhoid conjugate vaccines (TCV) offer a safe and effective intervention that can save lives.

Typhoid fever is common in South and South-East Asia and sub-Saharan Africa, while paratyphoid fever is prevalent in South and South-East Asia. The Global Burden of Disease (GBD) study estimated that there were 9.24 million (95% uncertainty interval [UI], 5.94–14.1) cases of typhoid fever in 2019 resulting in 110 000 (95% UI, 52 800–191 000) deaths [1]. GBD 2017 estimated 3.39 million (95% UI, 2.67–4.18) cases of paratyphoid fever and 19 108 (95% UI, 8706–37 332) deaths globally, with incidence reportedly increasing [2]. Children younger than 5 years are disproportionately impacted by enteric fever, highlighting the need for vaccination programs targeting this population. The emergence of antimicrobial resistant strains of *S.* Typhi and *S.* Paratyphi, especially extensively drug-resistant strains of *S*. Typhi, threatens treatment options and further supports TCV introduction [3]. In 2017, the WHO Strategic Advisory Group of Experts (SAGE) recommended the use of TCV in endemic countries [4]. There is still, however, no licensed paratyphoid vaccine.

## Ty21a AND Vi-POLYSACCHARIDE VACCINES

While Ty21a and Vi-polysaccharide vaccines were recommended by the WHO SAGE in 2008 for use in endemic countries, they were not adopted for widespread public health use. Estimates of oral Ty21a efficacy vary with the formulation, number of doses, and population tested. In field trials among children in Santiago, Chile, for example, 3 doses of vaccine given as an enteric-coated capsule yielded an efficacy of 67% over 3 years of follow-up and 62% after 7 years of follow-up against culture-confirmed illness [5]. Ty21a is recommended in a 3- or 4-dose schedule and is licensed for adults and children aged 6 years and older. The capsule formulation is difficult for young children to swallow. A single dose of parenteral Vi-polysaccharide prevents 64%–72% of blood-culture–confirmed typhoid fever 1–2 years after vaccination [5]. As with other polysaccharide vaccines, immune response is poor in young children and infants, and hence it is licensed for use in adults and children aged 2 years and older. The protection offered by the vaccine is short-lived and it fails to induce immunological memory.

## TYPHOID CONJUGATE VACCINES ARE AVAILABLE FOR GLOBAL PUBLIC HEALTH USE

The new generation of TCVs overcomes many limitations of earlier vaccines. Conjugating Vi-polysaccharide conjugated to a protein carrier induces a T-cell–dependent immune response, leading to a longer duration of protection, induction of memory responses, and improved immunogenicity in children younger than 2 years. Two doses of a Vi-polysaccharide conjugated to recombinant exoprotein A from *Pseudomonas* aeruginosa (Vi-rEPA, not licensed) demonstrated high protective efficacy, strong and long-lasting immunogenic response, and elicited immune response in young children in a large phase 3 trial in Vietnam [6]. TCVs have the potential to pave the way for development of more conjugate vaccines.

There are currently 2 WHO-prequalified TCVs. Typbar TCV, manufactured by Bharat Biotech, Hyderabad, India, uses tetanus toxoid (Vi-TT) as a carrier protein and was the first prequalified TCV [7]. Safety and immunogenicity of the vaccine was based in infants aged older than 6 months, children, and adults in a prelicensure phase 3 trial in India [8]. In a controlled human infection model in Oxford, UK, Typbar TCV achieved a vaccine efficacy of 54.6% in typhoid-naive adults using a stringent case definition [9]. Based on a field definition of typhoid fever, the study demonstrated high protection against clinical disease, comparable to Vi-rEPA. Further field trials conducted by the Typhoid Vaccine Acceleration Consortium (TyVAC) in Nepal, Malawi, and Bangladesh are generating additional evidence in support of Typbar TCV [10–12]. In Nepal, interim analyses of a double-blind, randomized trial in Nepal in children aged 9 months to 15 years achieved over 81% efficacy and immunogenicity in an endemic setting [13], confirming TCV is safe and protects against typhoid in children as young as 9 months.

TYPHIBEV, manufactured by Biological E, India, received WHO prequalification in December 2020 after demonstrating safety and immunogenicity comparable to Typbar TCV [14]. TYPHIBEV consists of the Vi-polysaccharide derived from *Citrobacter* conjugated to a mutant nontoxic diphtheria toxoid carrier protein, CRM_197_ [15, 16]. WHO prequalification of TYPHIBEV is good news for the global typhoid agenda. Two prequalified TCVs improves supply, especially for procurement through Gavi, the Vaccine Alliance and UNICEF, for introduction in endemic countries these life-saving vaccines.

## TCVS LICENSED, AWAITING LICENSURE, AND IN THE PIPELINE FOR PREQUALIFICATION

More TCVs are in various stages of development and licensure (Table 1). Availability of multiple vaccines is crucial for a healthy global market and to meet country demands. PedaTyph (Bio-Med, India), a Vi-TT, is licensed for use in India; however, it is not prequalified due to the lack of necessary data [17]. ZyVAC TCV (Cadila Healthcare Ltd, India), another Vi-TT, has demonstrated noninferiority to Typbar TCV and is licensed in India [18]. Vi-DT, containing Vi-polysaccharide conjugated to diphtheria toxoid, was developed by the International Vaccine Institute, Seoul, Republic of Korea [19]. The technology has been transferred to SK Bioscience (South Korea), PT Bio Farma (Indonesia), and Incepta Vaccines (Bangladesh). Phase 1 and 2 trials conducted by SK Bioscience and PT Bio Farma have shown promising results [20, 21]. SK Bioscience conducted a phase 3 trial in participants 6 months to 45 years old in Nepal, which confirmed the immune response is not inferior to Typbar TCV, and another phase 3 study is nearing completion in the Philippines [22]. SK Biosciences plans to apply for authorization from the national regulators followed by submission for prequalification review.

**Table 1.** Status of Typhoid Conjugate Vaccines (TCVs)

Vaccine	Description	Trade Name	Manufacturer	Status
Vi-rEPA	*Salmonella* Typhi Vi conjugated to recombinant exoprotein A from *Pseudomonas aeruginosa*	…	National Institutes of Health, USA	Technology transferred to Lanzhou Institute of Biological Product, China
		…	Lanzhou Institute of Biological Product, China	Phase 3 trial
Vi-TT	*S.* Typhi Vi conjugated to tetanus toxoid	Typbar TCV	Bharat Biotech International, Ltd, India	WHO prequalified, 2017
		PedaTyph	Bio-Med (P), Ltd, India	Licensed in India
		ZyVAC TCV	Cadila Healthcare, Ltd, India	Licensed in India
Vi-CRM_197_	*Citrobacter* Vi conjugated to nontoxic mutant of diphtheria toxin	…	GSK Vaccines Institute for Global Health	Technology transferred to Biological E, Ltd, India
		TYPHIBEV	Biological E, India	WHO prequalified, 2020
Vi-DT	*S.* Typhi Vi conjugated to diphtheria toxoid	…	International Vaccine Institute, South Korea	Technology transferred to SK Bioscience, South Korea, PT Bio Farma, Indonesia, and Incepta Vaccines, Bangladesh
		…	SK Biosciences, South Korea	Phase 3 trial
		…	PT Bio Farma, Indonesia	Plans underway for phase 3 trial
		…	Incepta Vaccines, Bangladesh	Preclinical

## COUNTRY-LEVEL IMPLEMENTATION OF TCV

In October 2017, WHO SAGE recommended the introduction of TCV in typhoid-endemic countries and in countries with a high burden of antimicrobial-resistant *S.* Typhi as a part of routine immunization programs at 9 months or 2 years of age, with catch-up vaccination up to 15 years of age, if feasible and supported by epidemiological data [4]. In line with this recommendation, Gavi announced support for TCVs in November 2017 and opened a funding window for 2019–2020. In less than 2 years, 3 countries have introduced TCV: Pakistan introduced TCV into routine immunization in November 2019; and Liberia introduced TCV with a campaign followed by routine immunization in April 2021; and, Zimbabwe launched a large integrated national vaccination campaign with TCV, human papillomavirus vaccine, and inactivated polio vaccine in May 2021.

Countries need burden and antimicrobial-resistance data to make informed decisions to prioritize TCVs; however, typhoid-endemic countries often lack the necessary data and are challenged to fill data gaps. Surveillance is essential to understand the country-specific disease burden—including incidence, mortality, and hospital admissions—associated with typhoid fever. In addition, many endemic countries lack the capability or capacity for blood culture-confirmation of cases, the gold standard for the diagnosis of typhoid fever as recommended by the WHO [22]. The Widal agglutination test, which is more widely used in endemic settings, is not reliable and the accuracy of available point-of-care rapid diagnostic tests is unclear [23].

Modelling data on country risk, and alternative outcomes, such as typhoid perforation, may be important surrogates to inform decisions in areas without the capacity for blood culture surveillance. Likewise, continued surveillance in those countries with the capacity is crucial and, with the heterogenicity of the disease, expanding surveillance to subnational and rural areas can inform decision making. Equally, it is important to identify the risk factors for typhoid fever and risk groups. This evidence can inform decisions on introduction, to identify the vaccine target group, and design the most appropriate the vaccine strategy to implement (universal vaccination vs phased vaccination and/or a risk-based approach). Establishing surveillance systems will be essential in the long run to evaluate the program after vaccine introduction.

Policy makers need to weigh the costs and benefits of introducing TCV, and the potential short- and long-term impact on the national health budget. Considerations for implementation include age of TCV introduction into routine immunization and for a catch-up campaign, if needed, as well as the target population. A cost-effectiveness analysis has shown that routine immunization at 9 months with catch-up campaigns through 15 years is optimal for Gavi countries that choose to introduce the vaccine [24]. Routine vaccination coupled with catch-up is likely to accelerate and lead to a more sustained decrease in the typhoid burden. Vaccination strategy, however, will depend on a country’s willingness to pay. Vaccination is most likely to be cost-effective in countries with ≥ 300 typhoid cases/100 000 [24]. The extent of antimicrobial resistance is likewise an important consideration.

## PARATYPHOID VACCINES AND BIVALENT VACCINES

The incidence of paratyphoid fever is rising, especially in South Asia, with *S.* Paratyphi A the most common serovar. In contrast, paratyphoid fever is virtually absent in Africa. There are currently no vaccines available to prevent paratyphoid, although there are conjugate vaccine candidates under development for paratyphoid fever alone as well as combination vaccine candidates for typhoid and paratyphoid fever. Paratyphoid vaccines alone are unlikely to be used at a national level; however, bivalent vaccines can potentially address the dual burden of typhoid and paratyphoid fever in countries where both pathogens are endemic.

There are several monovalent *S*. Paratyphi A vaccines and bivalent *S.* Paratyphi A–*S.* Typhi vaccines in development (Table 2). A single dose of lipopolysaccharide O-antigen (O:2), the protective antigen of *S*. Paratyphi A, conjugated to tetanus toxoid (developed by the United States National Institutes of Health and transferred to Chengdu and Lanzhou Institutes of Biological Products, China) has been found to be safe and immunogenic in phase 1 and 2 trials in adults, teenagers, and children aged 2–4 years [25]. Further phase 2 trials are ongoing with the target formulation a bivalent vaccine. *S.* Paratyphoid A conjugate vaccine using O:2 antigen conjugated to CRM_197_ (developed by GSK Vaccines Institute for Global Health) has been shown to be immunogenic [26]. The technology has been transferred to Biological E (India) and is intended for a bivalent vaccine combined with Vi-CRM_197_. A phase 1 trial of a live attenuated, oral vaccine candidate against *S*. Paratyphi A (CVD 1902), developed by the Center for Vaccine Development and Global Health at the University of Maryland School of Medicine, in adult volunteers, found the vaccine to be safe and immunogenic [27]. CVD 1902 has been licensed to Bharat Biotech (India), which also has a licensed live attenuated oral *S.* Typhi vaccine strain that could be combined in a bivalent vaccine.

**Table 2.** Status of *Salmonella* Paratyphoid A Vaccines

Vaccine	Description	Vaccine Manufacturer	Clinical Trial Stage	Bivalent Vaccine
Conjugated				
O:2-TT	O-specific polysaccharide (O:2) conjugated to tetanus toxoid	Developed by National Institutes of Health, USA Technology transfer to Chengdu and Lanzhou Institutes of Biological Products, China	Phase 2	Intended for a bivalent vaccine O:2,12-TT + Vi-TT
O:2-CRM_197_	O-specific polysaccharide (O:2) conjugated to carrier protein CRM_197_, a nontoxic mutant diphtheria toxin	Developed by GSK Vaccines Institute for Global Health Technology transfer to Biological E, Ltd, India	Preclinical	Intended for a bivalent vaccine O:2,12-CRM_197_ + Vi-CRM_197_
Live Attenuated				
CVD1902	Deletions in guaBA operon and clpX operon of *S*. Paratyphi A strain ATCC 9150	Developed by University of Maryland Center for Vaccine Development and Global Health (CVD) Licensed to Bharat Biotech International, Ltd, India	Phase 1	Intended for a bivalent vaccine CVD 1902 + CVD 909

Late-phase vaccine development is complicated by the low attack rate, which makes efficacy trials difficult. A controlled human infection model of *S*. Paratyphi has been developed by the University of Oxford [28]. Such a model can potentially be a valuable and cost-effective way to assess the efficacy of the current paratyphoid vaccine candidates to expedite vaccine development and implementation.

As with typhoid, it will be important to monitor the burden of paratyphoid fever, especially with implementation of TCV. Antimicrobial resistance against *S.* Paratyphi remains a threat and surveillance should be strengthened. Further, the cost-effectiveness of the paratyphoid and bivalent vaccine over other preventive measures will need to be explored to prioritize the vaccine for global use.

## CONCLUSION

Typhoid fever remains a major cause of morbidity and mortality in low- and middle-income countries, with evidence of a significant burden in children. With the prequalification of TCVs and Gavi funding, endemic countries have an opportunity to incorporate TCV into their national immunization programs, but more work is still needed to control paratyphoid fever.

## Notes

***Acknowledgment.*** We thank Leslie Jamka for her editorial revision of the manuscript.

***Financial support.*** This work was supported by the Bill and Melinda Gates Foundation (grant number OPP1151153 to the Typhoid Vaccine Acceleration Consortium [TyVAC]). TyVAC is a partnership between the Center for Vaccine Development and Global Health at the University of Maryland School of Medicine, the Oxford Vaccine Group at the University of Oxford, and PATH, an international nonprofit.

***Potential conflicts of interest.*** A. J. P. is Chair of the DHSC JCVI; a member of the WHO’s Strategic Advisory Group of Experts (SAGE); an NIHR Senior Investigator; and is chief investigator on clinical trials of Oxford University’s COVID-19 vaccine funded by NIHR. Oxford University has entered a joint COVID-19 vaccine development partnership with Astra Zeneca. K. M. N. is a member of the WHO’s SAGE. M. S. reports no potential conflicts of interest.

Presented in part: Asian Conference of Diarrhoeal Disease and Nutrition 28–30 January, 2020, Dhaka, Bangladesh.
